# Neutrophil-to-lymphocyte ratio and platelet-to-lymphocyte ratio as prognostic factors in patients with colorectal cancer: a retrospective study

**DOI:** 10.3389/fmed.2025.1630125

**Published:** 2026-01-13

**Authors:** Mohammad Sami El Muhtaseb, Layan Nidal Ismail, Tala A. Haddad, Huthifa Ghanayem, Shrouq Qudah, Daoud O. Al Aruri, Mohammad Abufaraj

**Affiliations:** 1 Department of General Surgery, School of Medicine, The University of Jordan, Amman, Jordan; 2 School of Medicine, The University of Jordan, Amman, Jordan; 3 Department of General Surgery, Jordan University Hospital, The University of Jordan, Amman, Jordan; 4 Department of Special Surgery, Division of Urology, School of Medicine, The University of Jordan, Amman, Jordan; 5 Department of Urology, Medical University of Vienna, Vienna, Austria

**Keywords:** colorectal cancer, inflammation, aging, neutrophil-to-lymphocyte ratio, platelet-to-lymphocyte ratio

## Abstract

**Background:**

Colorectal cancer (CRC) is a leading cause of cancer-related deaths worldwide. Neutrophil-to-lymphocyte ratio (NLR) and platelet-to-lymphocyte ratio (PLR) have been investigated as potential prognostic biomarkers. This study aims to evaluate the association between NLR and PLR with CRC across age groups and assess their prognostic value for overall survival (OS) and cancer-specific survival (CSS).

**Materials and methods:**

This is a retrospective study conducted on 285 CRC patients who underwent elective colectomy. Sociodemographic and clinicopathological characteristics were collected from electronic records. Preoperative NLR and PLR were calculated from blood samples. Descriptive analyses, multivariable logistic regression analyses, and survival analyses using Cox regression and Kaplan–Meier tests were used.

**Results:**

The highest median NLR was 4.54 and found among the youngest age group (<50 years). High NLR, defined as a value ≥3.19, was associated with worse OS (HR 2.23, *p* = 0.02) and CSS (HR 2.9, *p* < 0.05). After adjusting for confounding factors, high NLR remained significantly associated with worse CSS (adjusted HR = 2.89, *p* = 0.02). PLR did not show any significant associations with OS or CSS. Age, smoking status, and BMI were also independent prognostic factors for OS.

**Conclusion:**

High NLR was identified as an independent prognostic factor for worse CSS in CRC patients. The association between NLR and PLR with CRC outcomes may vary across different age groups. These findings highlight the potential utility of NLR as a prognostic biomarker in CRC, particularly for CSS.

## 1 Introduction

Worldwide, the prevalence of colorectal cancer (CRC) has been increasing (1). It is currently considered the third most commonly diagnosed cancer and the second most common cause of cancer death accounting for 9.4% of all cancer-related deaths (2). Surgical resection remains the only curative treatment option for the majority of colorectal cancers. However, a considerable proportion of CRC patients experience recurrence or metastasis within 5 years of surgery (3).

Several factors have been identified as predictors of recurrence and prognosis among CRC patients. These include tumor-specific factors and patient-related factors (4). Of these, the presence of metastasis, the volume of liver metastasis, and the nodal stage have been shown to be independent prognostic factors for survival (4). Beyond these variables, inflammation has emerged as a key factor in the development of cancers.

The link between inflammation and cancer, first identified by Rudolf Virchow in the 19th century (5), has been well established, with inflammation playing a significant role in tumor development and progression (6, 7). This response often manifests as abnormalities in blood components, particularly neutrophils, lymphocytes, and platelets. Among the various inflammatory markers, neutrophil-to-lymphocyte ratio (NLR) and platelet-to-lymphocyte ratio (PLR) have been demonstrated to influence clinical outcomes in cancers, including colorectal cancer (CRC). Studies that assessed the utilization of pre-operative NLR as a prognostic biomarker of CRC showed that high NLR correlated with poor clinical outcomes; both overall survival (OS) and cancer-specific survival (CSS) (8). Similarly, PLR is increasingly being evaluated in different diseases, with high platelet count being considered a poor prognostic indicator for several cancers, including CRC (9). PLR has been shown in numerous studies to be associated with CRC progression, patients’ outcomes, and survival (10).

The association between these inflammatory markers and age had been identified by several studies, demonstrating a correlation between higher levels, especially NLR, and advanced age (11, 12). However, the link with age in specific disease groups, especially younger patients and notably in CRC, remains limited. Therefore, the primary aim of this study is to evaluate NLR and PLR across different age groups and to assess their prognostic values regarding OS and CSS among patients with CRC.

## 2 Materials and methods

### 2.1 Study design and population

This was a retrospective study conducted on patients diagnosed with CRC who underwent elective colectomy between November 2012 and January 2022 at JUH; a tertiary hospital located in Amman, the capital city of Jordan. The data was collected from the Electronic Health Records (EHR), reviewed and analyzed retrospectively. The inclusion criteria were as follows: (1) all patients have undergone elective surgery, (2) CRC was confirmed by histopathology reports, and (3) the accessibility to complete peripheral blood counts. The exclusion criteria included: (1) immunocompromised patients, (2) patients with hematological disease or chronic inflammatory disease.

Patients with known chronic inflammatory or autoimmune conditions, immunodeficiency syndromes, or long-term use of immunosuppressive medications were excluded through manual review of patients’ documented diagnoses, medication history, and clinical notes in the electronic medical records.

Sociodemographic features (age at the time of diagnosis, gender, smoking status), clinicopathological features [tumor site, tumor stage, type of surgery performed, history of chemotherapy and non-steroidal anti-inflammatory drugs (NSAIDs) use, as well as height and weight for body mass index (BMI) calculation were obtained]. Right-sided CRC described cecal cancer, ascending colon cancer, and transverse colon cancer, while left-sided CRC described the descending colon, sigmoid, and the rectum. Elective colorectal resections included right hemicolectomy, left hemicolectomy, anterior resection, and total/subtotal colectomy. Tumor staging was performed according to the 8th edition of the Union for International Cancer Control-American Joint Committee on cancer classification for CRC.

Preoperative complete blood count (CBC) results, obtained routinely within 1 week prior to surgery, were retrieved from patients’ electronic medical records. These values were used to calculate the NLR and PLR, defined as the absolute neutrophil or platelet count divided by the absolute lymphocyte count, respectively.

No standardized follow up was carried out due to the retrospective nature of the study. The OS was interpreted from the date of surgery to the date of death of any cause or the last date of follow-up whereas CSS was defined as the time between the date of surgery to cancer-related death. The dates of death were extracted from the Jordan Civil Status and Passports Department (CSPD). Patients who were alive at last follow-up or lost to follow-up were treated as censored at the date of their last documented clinical encounter. Patients with missing follow-up data or incomplete laboratory records required for NLR and PLR calculation were excluded from the final survival analysis.

### 2.2 Statistical analyses

Receiver operating characteristics (ROC) analysis was used to determine the optimal cut point values for NLR and PLR. Area under the curve (AUC) and 95% confidence interval (CI) were calculated using CSS as a primary outcome. The sensitivity and specificity of each cut point were produced and the value with the maximum Youden index was determined as the optimal cutoff point.

Categorical variables were expressed using frequencies and percentages and continuous data using medians with interquartile range (IQR). Correlation of the variables with the NLR and PLR groups (high/ low) was done using Chi square test. Comparison of the median NLR and PLR across age groups and TNM stages was done using Kruskal-Wallis test. Kaplan Meier survival graphs were used to assess the correlation of NLR and PLR with the survival outcomes (OS/ CSS) and logrank test to assess the significance. Univariable Cox regression model was carried out to produce hazard ratios and 95% CI. All variables that reached statistical significance (*p* < 0.05) were adjusted for in the final multivariable regression model. Harrell’s Concordance index (C-index) was calculated to evaluate the performance of the model. *p* value < 0.05 was considered statistically significant. All analyses were done using Stata (StataCorp. 2015. Stata Statistical Software: Release 14. College Station, TX: StataCorp LP).

## 3 Results

For NLR and PLR, the optimal cutoff point was determined as 3.19 and 224.4, respectively. High NLR was defined as ≥3.19 and low NLR as <3.19. High PLR was defined as ≥224.4 and low PLR as <224.4.

### 3.1 Baseline characteristics

A total of 285 patients with histologically confirmed colorectal cancer were included in the final analysis. Fourteen percent of study participants were aged less than 50 years at the time of diagnosis, 45.6% were aged 50–69 years and 40% were in the age category 70 years or above. Male participants accounted for 55.8% of the study sample.

Right-sided and left-sided tumors were observed to have an approximately equal prevalence. As for the type of surgery performed, right hemicolectomy (40.7%) followed by left hemicolectomy (26.7%) were the most common. Regarding tumor characteristics, stage T3 was predominantly observed in 56.7% of the study population. Positive lymph node involvement was seen in 48.6% of patients while distant metastases was only found in 9.9% (Table 1).

**Table 1:** Clinical and pathological characteristics of 285 colorectal cancer patients.

Variable	Total, *n* (%)						
		NLR <3.19 (*n*, %)	NLR ≥ 3.19 (*n*, %)	*p*	PLR <224.4 (*n*, %)	PLR ≥224.4 (*n*, %)	*p*
Total	285 (100)	128 (44.91)	157 (55.09)		166 (58.25)	119 (41.75)	
Age at time of diagnosis (years)				0.03*			0.2
<50	41 (14.39)	15 (11.72)	26 (16.56)		20 (12.05)	21 (17.65)	
50–69	130 (45.61)	69 (53.91)	61 (38.85)		81 (48.8)	49 (41.18)	
≥70	114 (40)	44 (34.38)	70 (44.59)		65 (39.16)	49 (41.18)	
Gender				0.01*			0.6
Female	126 (44.21)	67 (52.34)	59 (37.58)		75 (45.18)	51 (42.86)	
Male	159 (55.79)	61 (47.66)	98 (62.42)		91 (54.82)	68 (57.14)	
Smoking history				0.5			0.7
Never	165 (62.98)	79 (66.39)	86 (60.14)		93 (61.18)	72 (65.45)	
Current smoker	78 (29.77)	32 (26.89)	46 (32.17)		47 (30.92)	31 (28.18)	
Former smoker	19 (7.25)	8 (6.72)	11 (7.69)		12 (7.89)	7 (6.36)	
NSAIDs				0.4			0.7
No	218 (84.17)	99 (82.50)	119 (85.61)		127 (83.55)	91 (85.05)	
Yes	41 (15.83)	21 (17.50)	20 (14.39)		25 (16.45)	16 (14.95)	
BMI (Kg/m^2^)				0.9			0.2
<25	113 (40.36)	50 (40)	63 (40.65)		60 (37.04)	53 (44.92)	
25–29.9	11 (39.64)	50 (40)	61 (39.35)		65 (40.12)	46 (38.98)	
≥30	56 (20)	25 (20)	31 (20)		37 (22.84)	19 (16.10)	
Site of tumor				0.5			0.6
Left side	142 (49.82)	61 (47.66)	81 (51.59)		81 (48.80)	61 (51.26)	
Right side	143 (50.18)	67 (52.34)	76 (48.41)		85 (51.20)	58 (48.74)	
Type of surgery				0.07			0.002*
Right hemicolectomy	116 (40.70)	53 (41.41)	63 (40.13)		70 (42.17)	46 (38.66)	
Left hemicolectomy	76 (26.67)	42 (32.81)	34 (21.66)		55 (33.13)	21 (17.65)	
Anterior resection	56 (19.65)	21 (16.41)	35 (22.29)		27 (16.27)	29 (24.37)	
Total/subtotal colectomy	37 (12.98)	12 (9.38)	25 (15.92)		14 (8.43)	23 (19.33)	
Primary tumor				0.026*			0.2
Tis	4 (1.41)	0 (0.00)	4 (2.56)		1 (0.60)	3 (2.54)	
T1	8 (2.82)	7 (5.47)	1 (0.64)		7 (4.22)	1 (0.85)	
T2	43 (15.14)	22 (17.19)	21 (13.46)		27 (16.27)	16 (13.56)	
T3	161 (56.69)	73 (57.03)	88 (56.41)		89 (53.61)	72 (61.02)	
T4	68 (23.94)	26 (20.31)	42 (26.92)		42 (25.30)	26 (22.03)	
Lymph nodes				0.5			0.6
Negative	137 (48.58)	59 (46.46)	78 (50.32)		78 (47.27)	59 (50.43)	
Positive	145 (51.42)	68 (53.54)	77 (49.68)		87 (52.73)	58 (49.57)	
Metastases				0.1			0.8
M0	256 (90.14)	118 (92.91)	138 (87.90)		150 (90.36)	106 (89.83)	
M1	28 (9.86)	9 (7.09)	19 (12.10)		16 (9.64)	12 (10.17)	
TNM stage				0.3			0.8
(T1–T4), N0, M0	128 (45.55)	57 (45.24)	71 (45.51)		73 (44.24)	55 (47.01)	
Any T, N (N1–N2), M0	126 (44.84)	60 (47.62)	66 (42.31)		76 (46.06)	50 (42.74)	
Any T, any N, M1	27 (9.61)	9 (7.14)	19 (12.18)		16 (9.70)	12 (10.26)	
Synchronous tumor				0.9			0.4
No	151 (57.41)	69 (57.50)	82 (57.34)		84 (55.26)	67 (60.36)	
Yes	112 (42.59)	51 (42.50)	61 (42.66)		68 (44.74)	44 (39.64)	
Chemotherapy				0.1			0.3
No	150 (62.50)	63 (57.27)	87 (66.92)		87 (60)	63 (66.32)	
Yes	90 (37.50)	47 (42.73)	43 (33.08)		58 (40)	32 (33.68)	

NLR, Neutrophil-to-lymphocyte ratio; PLR, Platelet-to-lymphocyte ratio; NSAIDs, non-steroidal anti-inflammatory drugs; BMI, body mass index. *Statistically significant, *p* < 0.05.

### 3.2 Association of NLR and PLR with clinical and pathological features

Depending on the NLR and PLR values, patients were categorized into four predefined groups; (1) Low NLR (*n* = 128, 44.9%), (2) high NLR (*n* = 157, 55.1%), (3) Low PLR (*n* = 166, 58.3%), (4) High PLR (*n* = 119, 41.8%). Age at time of diagnosis, gender and the primary tumor (*p* < 0.05 for all) were all significantly associated with NLR while only the type of surgery performed (*p* < 0.01) showed significant association with PLR (Table 1).

### 3.3 Association of NLR and PLR with age at time of diagnosis and TNM stage

A statistically significant variance in the median NLR was observed across the age groups (*p* = 0.016). The highest median (IQR) NLR was 4.54 (2.55–8.8) and observed in those aged less than 50 years at the time of diagnosis. As for PLR, no statistical difference was found; however, the highest reported median value was 238 and similarly found in the age group < 50 years. NLR and PLR showed no significant difference across the TNM stages (Table 2).

**Table 2:** NLR and PLR median values stratified by age groups and TNM stage.

Variable	NLR, median (IQR)	*p*	PLR, median (IQR)	*p*
Age (at time of diagnosis)		0.016*		0.06
<50	4.54 (2.55–8.8)		238 (179.89–292.45)	
50–69	2.86 (1.85–5.84)		181.80 (125.92–277.58)	
≥70	3.56 (2.65–5.52)		204.63 (139.61–302.50)	
TNM stage		0.7		0.7
T (T1–T4), N0, M0	3.53 (2.16–5.96)		204.08 (145.12–284.02)	
any T, N (N1–N2), M0	3.31 (2.06–6.59)		192.15 (130.68–292.45)	
any T, any N, M1	4.22 (2.94–5.16)		190.61 (14.43–298.21)	

NLR, neutrophil-to-lymphocyte ratio; PLR, platelet-to-lymphocyte ratio; IQR, interquartile range. *Statistically significant *p* < 0.05.

### 3.4 Survival outcomes

**Figure 1:**
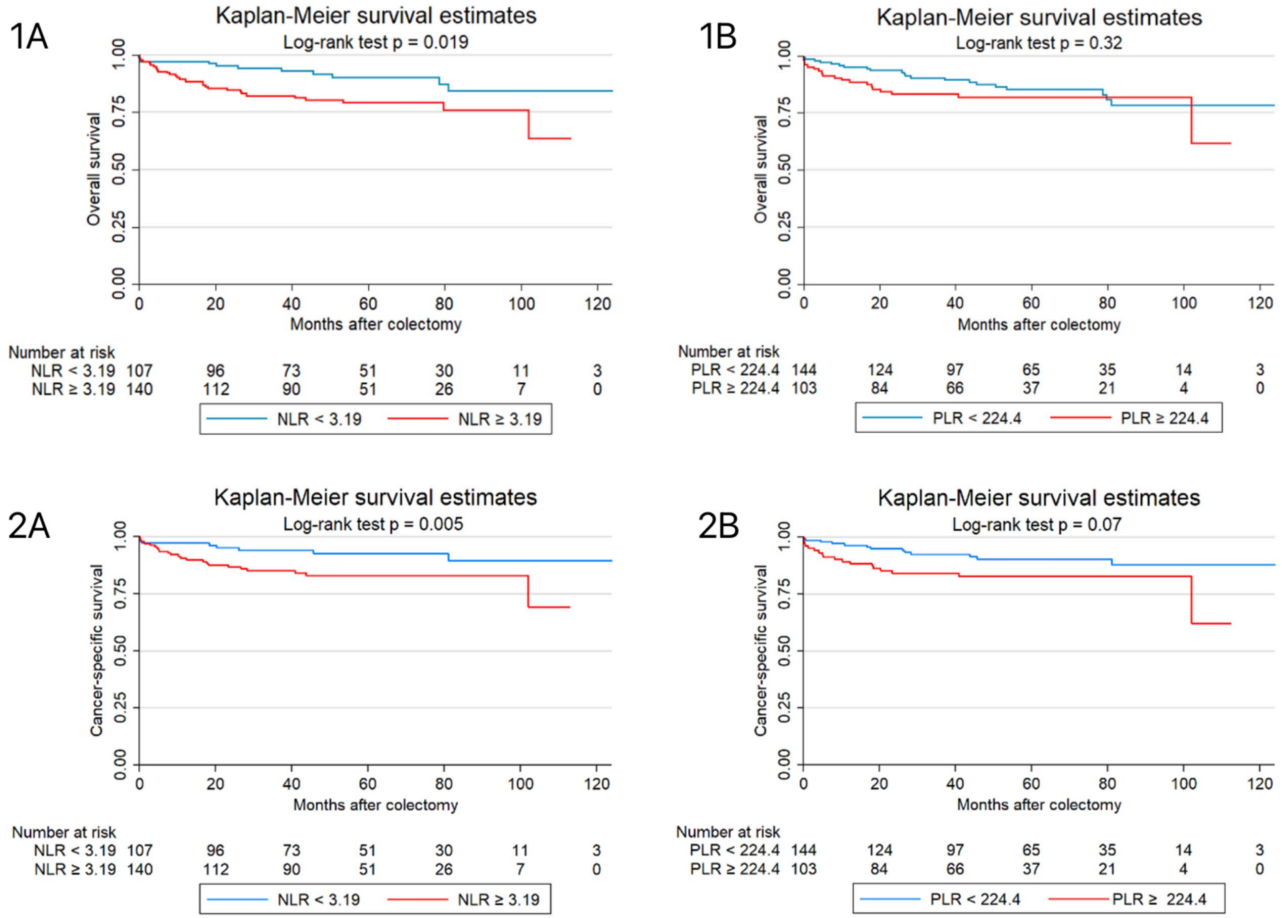
Kaplan–Meier analysis for overall survival (OS) and cancer-specific survival (CSS) among colorectal cancer patients who underwent colectomy. **(1A, 1B)** OS stratified by **(1A)** NLR at cutoff point 3.19 and **(1B)** PLR at cutoff point 224.4. **(2A, 2B)** CSS stratified by **(2A)** NLR at cutoff point 3.19 and **(2B)** PLR at cutoff point 224.4. NLR, neutrophil-to-lymphocyte ratio; PLR, platelet-to-lymphocyte ratio.

The median follow up was 58.9 months (IQR 41.43–80.26). Correlations of NLR and PLR with OS and CSS are graphically shown in Figure 1.

Poorer OS was significantly associated with high NLR (HR 2.23, *p* = 0.02), age above 50 years (HR 1.64, *p* = 0.001), current and former smoking history (HR 1.95, *p* < 0.01), and positive lymph node involvement (HR 1.98, *p* = 0.04). Higher BMI was observed to have an inverse significant association with OS (HR 0.6; *p* = 0.03). For OS, the multivariable model was adjusted for variables that yielded statistical significance of which age at time of diagnosis (HR 1.7, *p* = 0.001), smoking status (HR 2.02, *p* = 0.001), and BMI (HR 0.58, *p* = 0.03) remained significant (Table 3).

**Table 3:** Cox regression analysis of overall survival and cancer-specific survival.

Variable	Overall survival (OS), events observed (%) = 59 (20.7%)				Cancer-specific survival (CSS), events observed (%) = 41 (14.3%)			
	Univariable		Multivariable		Univariable		Multivariable	
	HR (95% CI)	*p*	HR (95% CI)	*p*	HR (95% CI)	*p*	HR (95% CI)	*p*
Age at time of diagnosis								
<50	Ref.		Ref.		Ref.		Ref.	
≥50	1.64 (1.23–2.19)	0.001*	1.7 (1.23–2.36)	0.001*	1.76 (1.28–2.42)	<0.001*	1.72 (1.20–2.45)	0.003*
Gender								
Female	Ref.				Ref.			
Male	1.84 (0.93–3.62)	0.07			1.53 (0.76–3.08)	0.2		
Smoking status								
Non-smoker	Ref.				Ref.			
Current/former smoker	1.95 (1.25–3.03)	0.003*	2.02 (1.31–3.12)	0.001*	1.4 (0.85–2.35)	0.1		
NSAIDs								
No	Ref.				Ref.			
Yes	1.02 (0.44–2.33)	0.05			1.06 (0.43–2.59)	0.8		
BMI								
<25	Ref.				Ref.			
≥25	0.6 (0.38–0.96)	0.03*	0.58 (0.34–0.96)	0.03*	0.68 (0.42–1.10)	0.1		
Site of tumor								
Left sided tumor	Ref.				Ref.		Ref.	
Right sided tumor	1.76 (0.92–3.34)	0.08			2.66 (1.27–5.55)	0.009*	2.27 (0.93–5.55)	0.07
Type of surgery done								
Anterior resection	Ref.				Ref.			
Right hemicolectomy	2.03 (0.81–5.05)	0.1			3.26 (0.96–11.12)	0.05		
Left hemicolectomy	0.97 (0.32–2.90)	0.9			1.31 (0.31–5.51)	0.7		
Total/subtotal colectomy	1.63 (0.52–5.08)	0.3			2.66 (0.63–11.14)	0.1		
NLR								
<3.19	Ref.		Ref.		Ref.		Ref.	
≥3.19	2.23 (1.11–4.48)	0.02*	1.91 (0.89–4.07)	0.09	2.9 (1.32–6.44)	0.005*	2.89 (1.13–7.39)	0.026*
PLR								
<224.4	Ref.				Ref.			
≥224.4	1.36 (0.73–2.54)	0.3			1.83 (0.94–3.57)	0.07		
Pathological T stage								
pT1	Ref.				Ref.		Ref.	
≥pT2	1.46 (0.91–2.34)	0.1			1.86 (1.11–3.11)	0.017*	1.10 (0.33–3.72)	0.8
Lymph node involvement								
Negative	Ref.		Ref.		Ref.		Ref.	
Positive	1.98 (1.02–3.82)	0.04*	1.68 (0.83–3.3)	0.1	2.5 (1.22–5.36)	0.013*	1.67 (0.39–7.03)	0.4
Metastases								
No	Ref.				Ref.		Ref.	
Yes	2.18 (0.95–4.97)	0.06			2.6 (1.13–6.08)	0.024*	2.59 (0.44–15.07)	0.2
Chemotherapy								
No	Ref.				Ref.			
Yes	1.04 (0.53–2.02)	0.9			0.79 (0.37–1.69)	0.549		
C index (without NLR)			0.818				0.760	
C index with NLR			0.825				0.770	

HR, hazard ratio; CI, confidence interval; NSAIDs, non-steroidal anti-inflammatory drugs; BMI, body mass index; NLR, neutrophil-to-lymphocyte ratio; PLR, platelet-to-lymphocyte ratio; C index, concordance index. *Statistically significant, *p* < 0.05.

For CSS, high NLR (HR 2.9, *p* < 0.01), older age at time of diagnosis (HR 1.76, *p* < 0.001), pathological T stage 2 and higher (HR 1.86, *p* = 0.01), positive lymph node involvement (HR 2.5, *p* = 0.01), right sided tumor (HR 2.66, *p* < 0.01), and presence of metastasis (HR 2.6, *p* = 0.02) were all significantly associated with worse CSS. Following the adjustment of significant confounding variables, only higher age at time of diagnosis (HR 1.72, *p* < 0.01), and high NLR (HR 2.89, *p* = 0.02) remained significant. PLR showed no statistical significance with OS or CSS (Table 3).

## 4 Discussion

The role of the immune system in the progression of various cancers, including CRC, has become increasingly recognized (13). This response can be evaluated by several serum markers, with the most robust being NLR and PLR, as they have been confirmed to have a significant role in predicting the prognosis of CRC (14). This study investigated the values of NLR and PLR among different age groups of CRC patients and assessed their prognostic value in regard to CSS and OS. Cutoff values of NLR and PLR have varied among studies, because articles presented different populations with varying tumor characteristics. However, NLR and PLR values ranged between 2 to 5 and 160 to 350, respectively, which aligns with our study (15).

When comparing NLR across age groups, two patterns emerged from our data. Older patients (≥70 years) demonstrated a statistically significant association with higher NLR values. However, median values categorized by age groups revealed that younger patients (<50 years) had the highest median NLR. Specifically, the median NLR in the <50 years group was 4.54, and the proportion of patients with high NLR (≥3.19) was also elevated (63.4%), comparable to the ≥70 years age group (61.4%). This paradox, where the youngest group has the highest median yet shares a similar proportion of high NLR with the oldest group, likely indicates different underlying mechanisms. In the younger age groups, this elevated median may be reflected by disproportionately high NLR in some individuals, which may represent a more aggressive disease course. Recent studies suggest that younger patients may have a unique subtype of CRC, with expanding literature relating elevated inflammatory markers to worse outcomes in this group (16, 17). However, further research is needed to confirm whether elevated NLR in early-onset CRC reflects a distinct immune-related phenotype with potential implications for prognosis and treatment. On the other hand, the association with older age groups reflects an overall trend when considering the entire age group distribution. These findings suggest that while older age is generally associated with higher NLR, specific outliers in younger patients may significantly influence the median, highlighting the complexity of interpreting NLR distribution across age groups. In comparison to other studies, Li et al. found that CRC patients with older age at diagnosis (>60) have significantly higher NLR (18). Other similar observations have also been published (11, 12, 19). These findings are consistent with the well-known effects of aging on increased inflammation (20), which can be confirmed by elevated NLR levels in older healthy patients (21). Importantly, although our age comparisons were descriptive in nature, the multivariate Cox models, which were adjusted for age, demonstrated that high NLR remained independently associated with cancer-specific survival, indicating that these age-related differences do not diminish its overall prognostic relevance. Regarding PLR, its associations with prognosis and age are still ambiguous. Similar to other studies, our findings showed no statistical relation between PLR levels and age (18, 22, 23). However, more research is needed to evaluate the relation between PLR and specific age groups.

When assessing other variables, depth of invasion, represented by pT stage, had a significant association with NLR. These findings are in alignment with other studies, where depth of invasion is frequently shown to have a significant association with inflammatory markers (11, 12, 18, 23–26). However, lymph nodes involvement (pN), Metastases (pM), and TNM stage did not show any significant relation with either NLR or PLR, with their association with these inflammatory markers still in debate (11, 12, 18, 23–26). In addition, the primary site of the tumor did not correlate significantly with either NLR or PLR. A discrepancy in the literature has been observed regarding the effect of tumor site on inflammatory markers, with some studies showing no influence on the levels (11, 27–29). However, PLR showed a significant association with the type of surgery performed, particularly in patients undergoing right hemicolectomy. The association between surgical approach and inflammatory markers like NLR and PLR may indicate that tumor location, rather than the surgery itself, is linked to the inflammatory response. This conveys the debate in literature regarding the relation between tumor location and inflammatory markers levels, as shown in our results, where tumor location itself did not have any significant association with markers levels.

While traditional prognostic factors such as TNM staging remain the cornerstone of CRC risk stratification, they are not always sufficient to accurately predict individual outcomes. According to that, inflammatory markers like NLR may provide prognostic values by reflecting the body’s immune response to tumor biology. Our findings support this by showing that NLR remained an independent predictor of CSS after adjusting for clinicopathological variables such as tumor stage, lymph node involvement, and metastasis. Although our dataset did not include other molecular biomarkers such as serum CEA, prior studies have demonstrated that elevated NLR correlates with poor outcomes even after controlling for these biomarkers (30, 31).

Regarding adjuvant chemotherapy, it was found to have no significant association with either NLR or PLR, which is in contrast to results published by Li et al., showing that patients who received adjuvant chemotherapy had significantly higher preoperative NLR and PLR levels compared to those who did not (18). This finding highlights that patients selected for adjuvant chemotherapy often had elevated inflammatory markers at baseline, reflecting more aggressive systemic inflammatory responses.

Our results also showed that patients with preoperative high NLR had poorer OS and CSS. Following the adjustment of confounding factors, high NLR remained significantly associated with worse CSS. In a systematic review of 71 studies, high NLR was prognostic of poor clinical outcomes regarding OS, comprising disease, recurrence and progression-free survival, which was in agreement with our current findings (8). The fact that NLR remained significantly associated with CSS, but not for OS, after multivariable adjustment reflects direct relation between systemic inflammation and tumor biology. In contrast, OS includes other non-cancer-related causes of death, which may dilute the cancer-specific prognostic value of NLR, particularly in older patients. Therefore, our findings suggest that NLR may function more accurately as a predictor of CSS rather than OS in CRC.

On the contrary, our study reported no association between PLR and OS or CSS, in contrast to previous studies where patients with high PLR possessed a significantly poorer OS (10). Additionally, PLR discriminatory capacity was limited in our cohort, which further reduces its clinical utility. These results suggest that routine use of PLR as a prognostic biomarker may not be useful in all CRC settings. Further large-scale, prospective studies are needed to evaluate whether PLR may hold predictive value in specific subgroups or treatment aspects.

Since NLR is calculated from a basic CBC, it comes at no extra cost as it is already part of routine preoperative assessments. Its availability makes it eligible for inclusion in risk stratification, especially in settings where advanced and expensive imaging, genomic studies, or tumor markers may not be available. In clinical practice, NLR could help identify patients who might need more intense follow-up, or consideration for adjuvant therapies including chemotherapy and radiotherapy. It may also be an early sign for high-risk cases. That said, major CRC guidelines, such as those from the NCCN and ESMO, have not yet included inflammatory markers like NLR or PLR in their risk models (32–34). However, growing evidence supports their prognostic value in preoperative risk stratification. Our findings add to the existing literature and propose that markers like NLR could one day become a useful part of standard prognostic models.

### 4.1 Limitations and strengths

Limitations of the study include the retrospective design. This was also a single institution study conducted in a single country. The patient’s sample size was limited. Although regression models were used to adjust for confounding variables, the risk of confounding bias cannot be totally excluded. Also, ethnic and regional differences may have an effect on baseline levels of inflammatory biomarkers. That said, such variations may impact the generalizability of our findings. While our results support the prognostic value of NLR in CRC, they should be investigated in multi-ethnic, multi-center cohorts to confirm external validation. Another limitation of our study is the lack of detailed treatment data other than chemotherapy. Information on radiotherapy or immunotherapy was not consistently available, which may have influenced survival outcomes or inflammatory marker profiles. Future prospective studies should account for these treatment variables to more accurately assess the prognostic value of NLR and PLR. On the other hand, one of the strengths of our study is that we used the absolute count of blood differentials to calculate NLR and PLR values without any approximation. Also, our inclusion of age-stratified analyses adds a detailed insight of how NLR differs across demographic groups, which may help modify risk assessment strategies for early vs. late-onset CRC.

## 5 Conclusion

In conclusion, the findings of our study indicate that preoperative NLR is an independent prognostic factor for CSS, with higher NLR significantly associated with poorer survival outcome. The feasibility of NLR calculation allows for simple biomarkers to be incorporated in colorectal cancer patients’ assessment preoperatively. We also observed meaningful variation in NLR across age groups, including disproportionately elevated values in a subset of younger patients, suggesting possible biological differences in early-onset colorectal cancer. Despite being consistently demonstrated across multiple studies, the role of NLR levels across different age groups should be studied more thoroughly to aid in the identification of patients with a more aggressive disease course at a younger age.

## Data Availability

The original contributions presented in the study are included in the article/supplementary material, further inquiries can be directed to the corresponding author.
